# PREVALENCE AND PREDICTORS OF INCREASED VERBAL/PHYSICAL CONFLICT DURING COVID-19: CLSA FINDINGS

**DOI:** 10.1093/geroni/igac059.109

**Published:** 2022-12-20

**Authors:** Gloria Gutman, Mojgan Karbakhsh, Heather Stewart

**Affiliations:** Simon Fraser University, Vancouver, British Columbia, Canada; Simon Fraser University Gerontology Research Centre, Vancouver, British Columbia, Canada; Simon Fraser University, Surrey, British Columbia, Canada

## Abstract

Child and spousal abuse rates have been shown to increase during various types of disasters. This study sought to determine the prevalence and determinants of older adults’ experiences of increased verbal or physical conflict (+VPC), as a proxy for elder abuse, during the COVID-19 pandemic. Data are from the Canadian Longitudinal Study on Aging (CLSA), a cohort study of 51,338 Canadians aged 45–85 at enrollment (2012-15) with follow-up every 3 years until 2033.. We analyzed data of participants aged 65 or above at follow-up1 who took part in a COVID-19 sub-study (n=24,306). Experiencing +VPC was the main outcome variable; explanatory variables included gender identity, sexual orientation, age group, race/ethnicity, educational attainment, marital status, household income, working status, living alone, social support availability, cohesion with community, self-rated physical and mental health, anxiety, depression, and previous history of elder abuse. The overall weighted prevalence of +VPC was 7.4%. Gay/bisexual men, 55-64 age-group, not living alone, low social support, poor social cohesion, low self-rated health, poor mental health, and past history of psychological or physical abuse were all significantly associated with +VPC. Weighted multivariable logistic regression revealed gender, not living alone, higher scores of depression and anxiety, and past history of psychological abuse to be independent predictors of +VPC. Implications for post-pandemic recovery and for prevention strategies during future disasters include targeted outreach programs for the most vulnerable group which includes males, persons age 55-64, those with self-rated poor mental health and/or history of elder psychological abuse.

